# The Effect of Cognitive Strategies and Facial Attractiveness on Empathic Neural Responses

**DOI:** 10.3390/ijerph192114617

**Published:** 2022-11-07

**Authors:** Michela Balconi, Natalia Kopiś-Posiej, Irene Venturella, Emilia Zabielska-Mendyk, Paweł Augustynowicz, Laura Angioletti

**Affiliations:** 1 International Research Center for Cognitive Applied Neuroscience (IrcCAN), Universita Cattolica del Sacro Cuore, 20123 Milan, Italy; 2 Research Unit in Affective and Social Neuroscience, Department of Psychology, Universita Cattolica del Sacro Cuore, 20123 Milan, Italy; 3 Department of Clinical Neuropsychiatry, Faculty of Medicine, Medical University of Lublin, 20-059 Lublin, Poland; 4 Department of Experimental Psychology, The Institute of Psychology, Faculty of Social Sciences, The John Paul II Catholic University of Lublin, 20-950 Lublin, Poland

**Keywords:** empathy for pain, empathy, facial attractiveness, pain_4_, neuroscience, EEG, ERP

## Abstract

Empathy is a phenomenon that brings together both emotions and an understanding of another person. Recent studies have disentangled the mechanisms of empathy into emotional and cognitive aspects. Event-related potential (ERP) studies suggest that emotional empathy is related to the modulation of the amplitude of early ERPs, and cognitive empathy is linked to later ERPs. In the current study, we examined the influences of facial attractiveness on empathic response and the effect of cognitive strategies with setting the participants’ attention to attractiveness or pain. Participants (N= 19) viewed photos of physically attractive and unattractive men and women receiving painful stimulation. The amplitude of the N2 component measured at the frontal regions was more negative in painful stimulation compared to the non-painful, but only for attractive faces. There were no differences between painful and non-painful stimulation for unattractive faces. The amplitude of the P3 measured at the central-parietal region component was more positive in the painful condition compared to the non-painful one, but only when participants performed a pain judgment task. There were no differences in the attractiveness judgment task. This study showed that the attractiveness of a model and drawing the participants’ attention to pain constitute an essential modulator of pain empathy.

## 1. Introduction

The possibility of empathizing with another person is a crucial ability to function properly in society. Empathy is defined as both sharing and understanding others’ feelings [1]. These two critical aspects of empathy are commonly referred to in the literature as emotional and cognitive empathy [2]. Emotional empathy allows one to identify and experience another person’s direct affective state [3]. In the case of cognitive empathy, it refers to the ability to take the perspective of another person in order to accurately understand their feelings [4]. These aspects allow distinguishing empathy from theory of mind (whose definition focuses on awareness and understanding other people’s internal states) and emotional contagion (where the observer is not aware of whether the source of emotions lies in him or the observed person) [5]. According to neuroscience research, the neuronal correlations of empathy include the activity of the brain structures that are active during the observation of affective experiences (mostly related to other people’s suffering) [5,6,7]. Thus, the perception of others’ faces (or body parts) in painful versus non-painful situations is common in empathy for pain research. Observing a person experiencing pain recruits the largely overlapping neural circuits compared to experiencing the pain directly. This neural empathic response is correlated with helping behavior and measures of empathy traits [8,9]. The emotional response to another person’s suffering occurs fairly reliably (at least for pain [10]). However, some studies have reported that it is not a static feeling. There are many factors that modulate both the brain’s responses and the subjective experience of empathy. These factors are related to appearance (e.g., [11]) or stereotypes associated with it (e.g., [12]). For example, a variable such as the human race was associated with a lack of neural activity in brain structures connected with the empathic response [12] or a lack of the differences in event-related potential (ERP) amplitudes, also interpreted as the index of empathy [11].

However, in the case of race, a few studies have attempted to modify this effect (e.g., by using cognitive strategies) [13]. In their successful attempt, Sheng and Han [13] decided to draw the participants’ attention to race or pain. The participants viewed Asian and Caucasian faces with neutral and painful facial expressions. A painful expression is characterized as a salient behavior, in any modality, which accompanies and is specific to pain. In Sheng and Han’s [13] experiment, the participants had to decide whether the person in the picture was of the same race as them(race judgment task) and whether the person in the picture felt pain or not (pain judgment task). There was a difference in ERP amplitude (P2 component) between pain and neutral expressions during race judgments, but only for the same race as a participant. However, during the pain judgment task, there were significant differences in the P2 component between the pain and neutral expressions for both races. Sheng and Han claimed that enhancing attention to an individual’s feelings (pain judgment task) increases empathy for others’ pain and reduces the influence of race on empathy.

Another important factor that can influence empathy is facial attractiveness. Judgements of physical attractiveness influence fertility, health, and gene quality [14]. It also plays a vital role in interpersonal evaluation such as marital satisfaction and socioeconomic status [15,16]. The definition of physical attractiveness can be formulated under the canon of beauty in today’s culture. However, compliance with the preferences of independent judges is a much simpler and often-used principle of assessing physical attractiveness (e.g., [17]). Facial attractiveness can be detected in a single glance or task unrelated to attractiveness judging, suggesting that it is an automatic process [18].

Additionally, physical attractiveness has been widely reported to impact human social behavior and interaction [19]. Several studies on empathy and attractiveness present in the literature have shown different results (see [20,21,22,23,24]). Some studies have claimed that viewing attractive models in painful situations influences a greater empathic response [21,23,24], while other studies have presented results in favor of unattractive ones [20,22,24]. In our previous study [25], we received an effect that may suggest that neural empathic response occurred when participants observed unattractive faces. The amplitude of the N2 ERP component localized in the frontal areas was significantly different for painful vs. non-painful stimuli, but only when participants observed unattractive faces. The N2 component may reflect the processes related to affective arousal in response to the observed stimulus [26]. Therefore, the difference in amplitude of this component suggests an unusual course of the process of sharing affect in empathy for pain. However, unlike most studies on empathy, in our experiment, participants had no other task except for memorizing the observed faces. It is speculated that a task unrelated to empathy (and related to memory) may have influenced the result obtained in the N2 component. It may also be related to the implicit processing of painful stimuli and the explicit processing of face features. Perhaps drawing the attention of the participants to faces favors the perception of the features, which are easier to memorize and, at the same time, enhance empathy for pain. Nevertheless, it is important to conduct a study that enhances the participants’ attention to an individual’s feelings by employing natural, color-scale pictures of people whose facial attractiveness was assessed by independent judges (in contrast to studies in [24]).

Therefore, the present study examined the cognitive strategies (see [13]) by introducing two tasks: the pain judgment task and the attractiveness judgment task. We specifically examined neural empathic responses over early processing stages (N2) and later cognitive stages (P3).

A recent meta-analysis [27] suggested that the later components were more reliable. In fact, the meta-analysis did not find significant effects of the N2; however, this may be due to the ambiguity of the direction of the effect, with some studies finding more negative mean amplitudes and others more positive when comparing pain versus no-pain (see [28]). In addition, the interaction between attractiveness and painful stimulation was registered in the N2 component in our previous study. In this study, we used faces with a neutral expression with the needle in the cheek (a painful stimulus) or with a Q-tip touching the cheek (non-painful stimuli). The same type of stimuli was used in our previous study [25,29] and other experiments (e.g., [11,30]). The amplitude of the N2 is sensitive to experimental modifications of variables (e.g., [11,31]). Its occurrence is interpreted as emotional empathy [32], and the attenuation of the N2 amplitude is due to the suppression of the affective response. The amplitude of the P3 component during empathy processing is interpreted as an index of the cognitive process of empathy, which allows us to consciously understand and accept another person’s perspective [2]. It is believed that the more positive amplitude of the P3 component “suggests that these stimuli are processed more deeply or fully” [33] (p. 130). However, the P3 component has also been shown to be an index of stimulus discrimination [34], implicated in orienting attention toward novel stimuli [35] and emotionally salient stimuli [36].

In the studies of Fan and Han [32], the amplitude of this component was higher for painful stimuli compared with non-painful ones, but only when participants had to perform pain judgment tasks. This result was repeated in other empathy for pain research (e.g., [29,37]). Furthermore, the P3 component reflects the evaluation or categorization of a stimulus supported by the observation that when the participants were instructed to perform the task accurately, the amplitude of P3 was higher than during performing it as fast as possible [38].

Considering the results from our previous study, we can assume that the empathic response (interpreted as the difference between painful and non-painful stimuli in the amplitude of the N2 component) will occur when participants see physically unattractive faces in a painful situation compared to a non-painful situation. However, participants may pay attention to different features during attractiveness and pain judgment tasks. Thus, it is possible that during the attractiveness judgment task, the empathic response will depend on physical attractiveness.

Additionally, the difference in the N2 component will only be present between painful and non-painful stimuli during pain judgment, independent of facial attractiveness. This hypothesis was based on the results of the study by Sheng and Han [13].

Furthermore, because the P3 component reflects the cognitive aspect of empathy, similarly as in Fan and Han [32], we hypothesized that the amplitude of this component would be higher when pain-related stimuli compared to neutral ones are presented, but only when the participant’s attention is drawn to pain (pain judgment). However, because P3 interprets it as an index of stimulus discrimination, we can assume that during the attractiveness judgment, the P3 amplitude might differ between attractive and unattractive models [39].

## 2. Method

### 2.1. Participants

Nineteen students (13 females; mean aged = 24.1, SD = 2.2; 6 males; mean aged = 25, SD = 3.3) were recruited through the Università Cattolica del Sacro Cuore in Milan, Italy. All participants were right-handed and in possession of normal or corrected-to-normal vision. All of the participants were volunteers who gave their written consent to participate in the study. The participants declared that they were not taking medication or other psychoactive substances on a permanent basis. The study was approved by the Research Team’s University Ethics Committee and was conducted in accordance with the Declaration of Helsinki.

### 2.2. Stimuli

The stimuli were taken from the Chicago Face Database [40] and the website www.models.com. A total of 268 photos were evaluated by 26 independent judges (14 females; mean age = 25.27; SD = 3.83) on scale 1-5 (1—very unattractive; 5—very attractive). The photographs were colorful, presented *en face*, with a size of 131.22 × 77.5 mm. For the electroencephalographic study, the four sets of 15 photos each were collected: attractive women (score: M = 4.04; SD = 0.14), attractive men (score: M = 3.9; SD = 0.26), unattractive women (score: M = 1.65; SD = 0.17), and unattractive men (score: M = 1.57; SD = 0.07). As a result, a total of 60 photographs were used. Each photograph was scaled to fit in a rectangular portion of the computer screen at a viewing distance of approximately 70 cm. Each face was manipulated digitally to be displayed in two different conditions. In the painful stimulation condition, the face was displayed with a syringe needle penetrating the cheek (right or left). In the non-painful stimulation condition, the face was displayed with a Q-tip touching the cheek (right or left) (for the sample of the stimuli, see [25,41]).

### 2.3. Procedure

Participants were informed that the experiment aimed to collect information about facial perception. They received instructions to perform two judgment tasks: to answer the question about pain (“Is this person feel pain?”) and to answer the question about attractiveness (“Is this person physically attractive?”). Participants were instructed to press either the right (“yes”) or left key (“no”) of the mouse to answer the question. These two tasks were organized in two blocks, counterbalanced for each participant. Each participant had to perform two blocks to complete the whole experiment.

**Figure 1:**
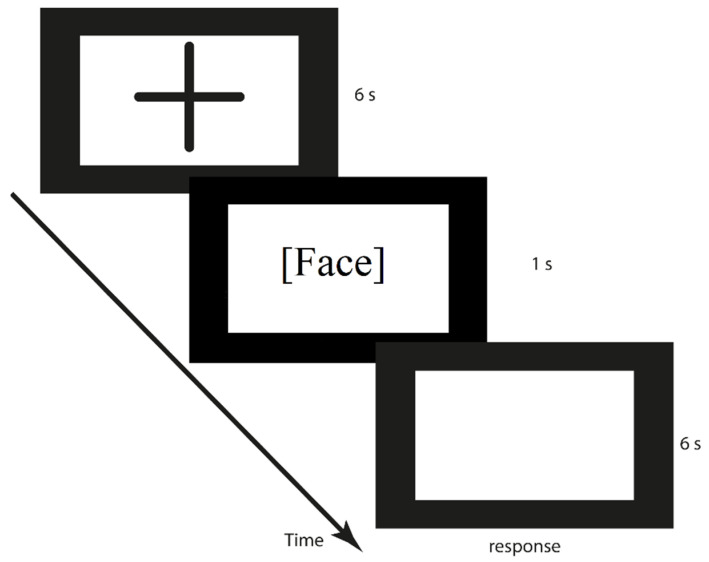
Illustration of the experimental procedure.

Each attempt started with a presentation of the fixation point in the middle of the computer screen by 6000 ms. Then, for 1000 ms, the face was presented, and an empty board was displayed for the next 6000 ms (Figure 1). It should be pointed out that we used functional near-infrared spectroscopy (fNIRS) except for the EEG equipment. This is why the breaks between pictures were longer than expected in the EEG procedure. There were 240 photos in total (30 attractive, 30 unattractive models, with balanced needle/Q-tip stimuli and left/right cheek touches), each presented in random order in the pain judgment block and attractiveness judgment block. In each block, all possible combinations of the face’s sex, attractiveness, and stimulation conditions were randomly ordered at run-time for each participant. The whole study (with the brakes) took 1.5 h.

### 2.4. EEG Acquisition and Analysis

For the signal acquisition, 19 active electrodes (AgCl; ActiCAP, Brain Products, Munich, Germany, 10–20 system) connected to a Compumedics NeuroScan SynAmps32 (Victoria, Australia) amplifier were used. The EEG was referenced to the average of the left and right mastoid electrodes and digitized at a sampling rate of 1000 Hz. Electrode impedances were kept below 5 kΩ. Offline signal processing included band-pass filtering (0.1 to 40 Hz). Muscle artifacts in the EEG signal including eye movements and eye blinks were corrected, and bad channels were removed using artifact subspace reconstruction method [42]. After that, an additional artifact rejection procedure was applied using independent component analysis (ICA; [43]) and source localization. We rejected independent components that contained remaining muscle artifacts, components whose source location was outside the brain, or that had unusual spectral power properties. Afterward, epochs were created, beginning at 200 ms before the onset of stimuli and ending 1000 ms after it with a pre-stimulus baseline correction. Epochs with abnormal (±70 µV) amplitude peaks were excluded from the analysis.

The N2 was analyzed using the average waveforms from the AF3, AF4, F3, F4, AF7, AF8 (frontal region) in a time window of 250–350 ms and the P3/LPP were analyzed using the average waveforms from CP3, CP4, CPz, Pz, P4, and P3 electrodes (central–parietal region); these regions of interest were based on those used by Galang, Jenkins, Obhi [28] and Xiong and colleagues [44].

The breakdown of the average number of valid trials per condition is reported in Table 1.

### 2.5. Statistical Analyses

The ERP data were analyzed using EEGLav v2020 to determine the differences in neural activity between the observation of attractive and unattractive faces in pain and neutral conditions for the task of judging the attractiveness and pain. A repeated-measures ANOVA was conducted for every mean ERP components’ amplitude of N2 (250–350 ms) on the frontal electrodes (AF3, AF4, F3, F4, AF7, AF8) and P3 (320–580 ms) on the centro-parietal electrodes (CP3, CP4, CPz, P3, P4, Pz), with within-subject factors: attractiveness (attractive vs. unattractive faces), stimulation (painful vs. non-painful stimuli), and cognitive strategies (pain vs. attractiveness judgment task). A level of *p* < 0.05 was used for all comparisons (Bonferroni corrected for multiple tests used for each ERP component), and all post hoc tests were further adjusted for multiple comparisons using Bonferroni correction.

## 3. Results

The response accuracies were high. The accuracy during pain judgment was 95.3%, and during attractiveness judgment it was 93.1%.

### 3.1. ERP: N2 (250–350 ms)

**Figure 2:**
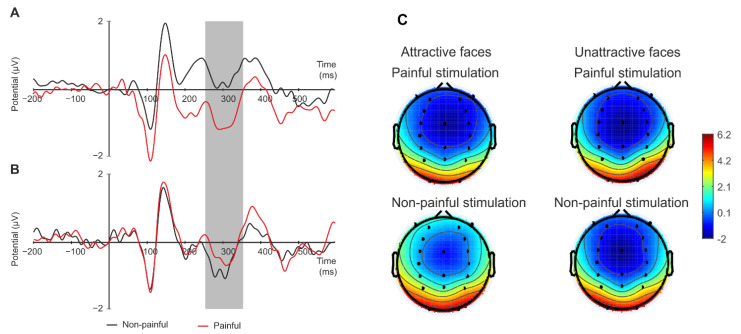
(**A**–**C**) N2 (250-350 ms), recorded at a selection of average frontal electrodes, relative to the two stimulation conditions (painful vs non-painful) for attractive faces (**A**) and unattractive faces (**B**). (**C**) Topographic maps of the N2 ERP component.

A two-way ANOVA with the attractiveness (attractive vs. unattractive faces), stimulation (painful vs. non-painful stimuli), and cognitive strategies (pain vs. attractiveness judgment task) as within-subject factors showed significant interaction of stimulation and attractiveness (F(1.18) = 7.181; *p* = 0.008; *p*ɲ^2^ = 0.33) proved to be significant. The Bonferroni post hoc test (*p* = 0.006) showed that the amplitude of component N2 was more negative in the painful stimulation condition (M = −0.83 μV; SE = 0.37 μV) compared to the non-painful (M = 0.24 μV; SE = 0.57 μV), but only for attractive faces. There were no differences between the painful and non-painful stimulation condition for unattractive faces (painful: M = −0.37 μV; SE = 0.47 μV; non-painful: M = −0.71 μV; SE = 0.49 μV) (Figure 2A–C). ANOVA detected no other main effect or interaction (all *p*s > 0.05).

### 3.2. ERP: P3 (320–580 ms)

**Figure 3:**
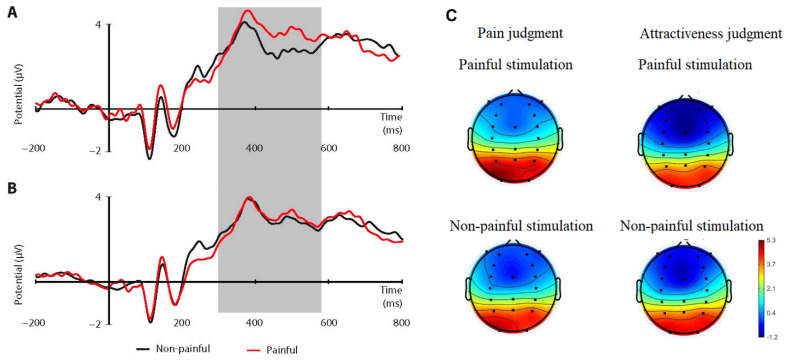
(**A**–**C**) P3 (320-580 ms), recorded at a selection of average centro-parietal electrodes relative to the two stimulation conditions (painful vs. non-painful) for the pain judgment (**A**) and attractiveness judgment task (**B**). (**C**) Topographic maps of the P3 ERP component (320-580 ms).

A two-way ANOVA showed that the interaction of stimulation and cognitive strategy (F(1.18) = 9.056; *p* = 0.008; *p*ɲ^2^ = 0.36) proved to be significant. The Bonferroni post hoc test (*p* = 0.003) showed that the amplitude of component P3 was more positive in the painful stimulation condition (M = 3.58 μV; SE = 0.54) compared to the non-painful one (M = 2.99 μV; SE = 0.59) but only when participants performed the pain judgment task. There were no differences during the block with the question about attractiveness (*p* = 0.51) (painful: M = 2.75 μV; SE = 0.31 μV; non-painful: M = 2.92 μV; SE = 0.28 μV) (Figure 3A–C). ANOVA detected no other main effect or interaction (all *ps* > 0.05).

## 4. Discussion

This study investigated the brain potentials related to the neural processing underlying empathy for pain, mainly facial attractiveness, and the effect of cognitive strategies. We found that the amplitude of the N2 ERP component was modified by the painful stimuli and physical attractiveness of the face. The N2 amplitudes during the processing of painful stimuli differed significantly from the N2 amplitudes during the processing of non-painful ones, but only in response to attractive faces. At the later stage of 320–580 ms, with P3 recorded on the centro-parietal electrodes, painful stimuli elicited a significantly stronger neural response than non-painful one, but only when the participants performed the pain judgment task. These results suggest that different empathic responses were present in earlier and later brain activity stages.

The difference in the amplitude of the N2 component between painful and non-painful stimuli, which appeared only for attractive faces, was independent of the cognitive strategy performed by the participants. This result was only partially congruent with our hypothesis. We claimed that facial attractiveness and cognitive strategy would influence the empathic reaction observed in the N2 component. While creating our experiment, we relied on Sheng and Han’s study [13], and we adopted a similar procedure and similar conceptual references (for instance, the reference to the term “cognitive strategies”). In their experiment, drawing the participants’ attention to pain reduced the racial effect. It was observed in the P2 ERP amplitude, which was higher for the painful facial expression compared with a neutral one for both races during the race judgment task.

It should be noted that in our experiment, we did not use stimuli with painful facial expression but faces stimulated with a needle. Faces accompanied by a stimulus causing pain (e.g., needle) seemed to be a more obvious tip for participants. The participant saw the needle pounded into the model’s cheek and knows that this can cause pain. This kind of stimulus does not require interpretation of the meaning, whereas the facial expression of pain is considered as a more subjective stimulus [45]. Observing the facial expression of pain demands a more analytical way of processing it because the participant has to interpret this expression as painful, which might not be so obvious. This could explain the results of Sheng and Han’s experiment [13], where different cognitive strategies caused differences in early ERP.

Additionally, the empathic response observed in the N2 component was interpreted as emotional empathy. Therefore, this component may “appear automatically”, as if the process of judging on facial attractiveness or painful stimulation may not influence the N2 amplitude. It should also be noticed that in other studies, the N2 component was present when observing the pain of others, regardless of the requirements of the task [32,46]. In most studies on ‘empathy for pain’ and the variables that can influence the empathic response (e.g., race), a procedure in which participants are required to assess the observed pain is used very often. However, it did not change the results observed in the amplitude of the N2 component. For example, there was still a difference between painful and non-painful stimuli that appeared only to the same race as the participant in the N2 component (e.g., [11]).

It should also be noted that the N2 component reflects the affective arousal associated with stimulus valence [26]. In the context of empathy, the reduced amplitude of this component may suggest an atypical course of the process of sharing affect in empathy toward pain for physically unattractive people. It should be noted, however, that the stimulus itself, which is used to inflict pain in the photos (e.g., a needle), can effectively attract the observer’s attention. As research has shown (see [47,48]), the stimuli that attract attention most effectively are essential for survival (e.g., stimuli related to reproduction or threatening stimuli). Combined with attractive faces that also draw the participant’s attention, this can make it easier to process the stimulus as an affective one and, at the same time, have an influence on empathic response.

Additionally, we found differences in the P3 component between painful and non-painful stimuli when the participants performed the pain judgment task. This result was congruent with our hypothesis and knowledge that the P3 component appears as a difference between painful and non-painful stimuli [11,30]. The occurrence of P3 was associated with the pain judgment task performed by the participants, which directed their attention to the observed pain. The cognitive strategy enhanced the individuated processing of persons and may increase references to an individual’s situation. At the same time, it affects a better understanding of the painful situation in which the observed person is in [11].

The P3 component is interpreted as the cognitive aspect of empathy, mostly connected with the conscious processing of information about pain. Nevertheless, the occurrence of the P3 component is also interpreted as an index of stimulus discrimination [34], implicated in orienting attention toward novel stimuli [35] and emotionally salient stimuli [36]. Therefore, we hypothesized that during the attractiveness judgment task, the amplitude of the P3 will differ for attractive and unattractive models. It is possible that in the case of the pain judgment task, the integration of the structural information of the faces (attractive and unattractive features) occurs, which is related to cognitive processes. Physical appearance does not influence our understanding of the painful situation (cognitive empathy) when our attention is drawn to another person’s suffering. However, during the attractiveness judgment, the participant has to again categorize the faces as attractive or unattractive. The combination of painful stimulation may impede attention-orienting processes (as suggested by the theory of metacognition, with specific reference to some control processes or attention regulation [49]). In addition, these processes may be represented as specific “strategic thinking” and also as a strategic form of emotions.

## 5. Conclusions

In summary, the results of this study suggest that facial attractiveness influences the early perceptual stages, but not the late cognitive stages of neural responses in empathy for pain. This may indicate that many positive attributes that surround attractive people cause us to see them in painful situations and share feelings with them (emotional empathy) to a greater extent. Additionally, these data confirm the previous literature thesis that proposes empathy as a mechanism constituted by both early, automatic processes and later, more cognitively controlled mechanisms [50].

## 6. Limitations

There are several limitations to the results of the present study. The study included 19 participants, and the group was not balanced. However, in the case of ERP studies, the parameters necessary for the correct estimation of the sample size (i.e., the value of variance or correlation between repeated measurements), are often not reported [51]. Because there is only one measurement (single EEG signal recording from one person), the number of stimuli in the test is repeated. Therefore, small group sizes are allowed in the ERP paradigm. Because ERP studies do not report the parameters needed to estimate the sample size correctly, the best approach is to rely on data from previous similar studies, as we have done in the present work. Moreover, because of the fNIRS, there were 240 trials, which limited the number of statistical comparisons.

In addition, we did not collect extensive information about the participants’ psychological health condition, and the present findings only represent the immediate effects in a laboratory setting. Caution should be exercised when generalizing. This is especially true in light of previous studies by Kopiś and colleagues [25] or Meng and colleagues [24]. Additional research on the relationship between attractiveness and empathy for pain is necessary. Although our experiment demonstrates the association between attractiveness and empathic neural response, we suspect that this relationship is not invariant. Future research should include the gender of the participant and the gender of the model as separate variables in the experimental design.

## Figures and Tables

**Table 1:** Breakdown of the average number of valid trials per condition.

M/SD	Pain Judgment				Attractiveness Judgment			
	Painful		Non-Painful		Painful		Non-Painful	
	Atr.	N.Atr	Atr.	N.Atr	Atr.	N.Atr	Atr.	N.Atr
M	29.68	29.05	29.16	28.89	29.26	29.37	29.36	28.68
SD	0.82	1.87	1.26	2.51	1.45	1.59	1.12	2.65

## Data Availability

The dataset generated for this study is available on request to the corresponding author.
